# Hydroxychloroquine lowers Alzheimer’s disease and related dementias risk and rescues molecular phenotypes related to Alzheimer’s disease

**DOI:** 10.1038/s41380-022-01912-0

**Published:** 2022-12-28

**Authors:** Vijay R. Varma, Rishi J. Desai, Sheeja Navakkode, Lik-Wei Wong, Carlos Anerillas, Tina Loeffler, Irene Schilcher, Mufaddal Mahesri, Kristyn Chin, Daniel B. Horton, Seoyoung C. Kim, Tobias Gerhard, Jodi B. Segal, Sebastian Schneeweiss, Myriam Gorospe, Sreedharan Sajikumar, Madhav Thambisetty

**Affiliations:** 1 Clinical and Translational Neuroscience Section, Laboratory of Behavioral Neuroscience, National Institute on Aging, Baltimore, MD USA; 2 Division of Pharmacoepidemiology and Pharmacoeconomics, Department of Medicine, Brigham and Women’s Hospital and Harvard Medical School, Boston, MA USA; 3 Lee Kong Chian School of Medicine, Nanyang Technological University, Singapore, Singapore; 4 Department of Physiology, National University of Singapore, Singapore, Singapore; 5 Healthy Longevity Translational Research Programme, Yong Loo Lin School of Medicine, National University of Singapore, Singapore, Singapore; 6 Laboratory of Genetics and Genomics, National Institute on Aging, National Institutes of Health, Baltimore, MD 21224 USA; 7 QPS Austria GmbH, Parkring 12, 8074 Grambach, Austria; 8 Center for Pharmacoepidemiology and Treatment Science, Ernest Mario School of Pharmacy, Rutgers University, New Brunswick, NJ USA; 9 Department of Medicine, Johns Hopkins University School of Medicine, Baltimore, MD USA; 10 Life Sciences Institute Neurobiology Programme, National University of Singapore, Singapore, Singapore

**Keywords:** Psychiatric disorders, Drug discovery

## Abstract

We recently nominated cytokine signaling through the Janus-kinase–signal transducer and activator of transcription (JAK/STAT) pathway as a potential AD drug target. As hydroxychloroquine (HCQ) has recently been shown to inactivate STAT3, we hypothesized that it may impact AD pathogenesis and risk. Among 109,124 rheumatoid arthritis patients from routine clinical care, HCQ initiation was associated with a lower risk of incident AD compared to methotrexate initiation across 4 alternative analyses schemes addressing specific types of biases including informative censoring, reverse causality, and outcome misclassification (hazard ratio [95% confidence interval] of 0.92 [0.83–1.00], 0.87 [0.81–0.93], 0.84 [0.76–0.93], and 0.87 [0.75–1.01]). We additionally show that HCQ exerts dose-dependent effects on late long-term potentiation (LTP) and rescues impaired hippocampal synaptic plasticity prior to significant accumulation of amyloid plaques and neurodegeneration in APP/PS1 mice. Additionally, HCQ treatment enhances microglial clearance of Aβ_1-42,_ lowers neuroinflammation, and reduces tau phosphorylation in cell culture-based phenotypic assays. Finally, we show that HCQ inactivates STAT3 in microglia, neurons, and astrocytes suggesting a plausible mechanism associated with its observed effects on AD pathogenesis. HCQ, a relatively safe and inexpensive drug in current use may be a promising disease-modifying AD treatment. This hypothesis merits testing through adequately powered clinical trials in at-risk individuals during preclinical stages of disease progression.

## Introduction

Advances in understanding the basic biology of Alzheimer’s Disease (AD) have not translated into effective treatments [1]. Traditional drug discovery approaches have focused on modifying either amyloid plaque or neurofibrillary tangle pathologies, which may be downstream events in a cascade that is initiated years before these pathologies appear. Therefore, identifying the earliest molecular abnormalities in disease progression may be key to developing effective treatments for AD. Equally importantly, there is growing consensus that pharmacological modulation of multiple key pathogenic pathways simultaneously may be preferable to agents against single targets [2].

We recently defined a hypothetical network of interacting and intersecting metabolic pathways in Alzheimer’s disease, linked to dysregulation in brain glycolysis- the Alzheimer’s Disease Aberrant Metabolism (ADAM) network [3]. We nominated genetic regulators of metabolic and signaling reactions in the ADAM network as plausible AD drug targets. Cytokine signaling through the Janus-kinase–signal transducer and activator of transcription (JAK/STAT) pathway was nominated as one such drug target for pharmacoepidemiologic analyses in the Drug Repurposing for Effective Alzheimer’s Medicines (DREAM) study [3]. Prior studies have suggested that dysregulation in this pathway may be associated with neurodegenerative diseases [4, 5] and may therefore may be a plausible therapeutic target [6–8]. We recently showed that disease modifying antirheumatic drugs (DMARDs) including tofacitinib (a JAK inhibitor), and 2) tocilizumab (an interleukin [IL]-6 inhibitor) were not associated with risk of AD and related dementias (ADRD) while TNF inhibitors may reduce risk of ADRD among patients with cardiovascular disease [9]. We additionally showed that C188-9, an experimental STAT3 inactivator currently in human clinical trials of cancer, rescued several molecular phenotypes relevant to AD in cell culture-based phenotypic assays [10].

Among existing FDA approved treatments, hydroxychloroquine (HCQ), a brain-penetrant DMARD, was recently shown to impact the JAK/STAT pathway through the inactivation STAT3 in lung adenocarcinoma cells suggesting a novel pharmacological approach in cancer chemotherapy [11]. We therefore hypothesized that HCQ may also impact AD pathogenesis and risk through the inactivation of STAT3.

**Fig. 1:**
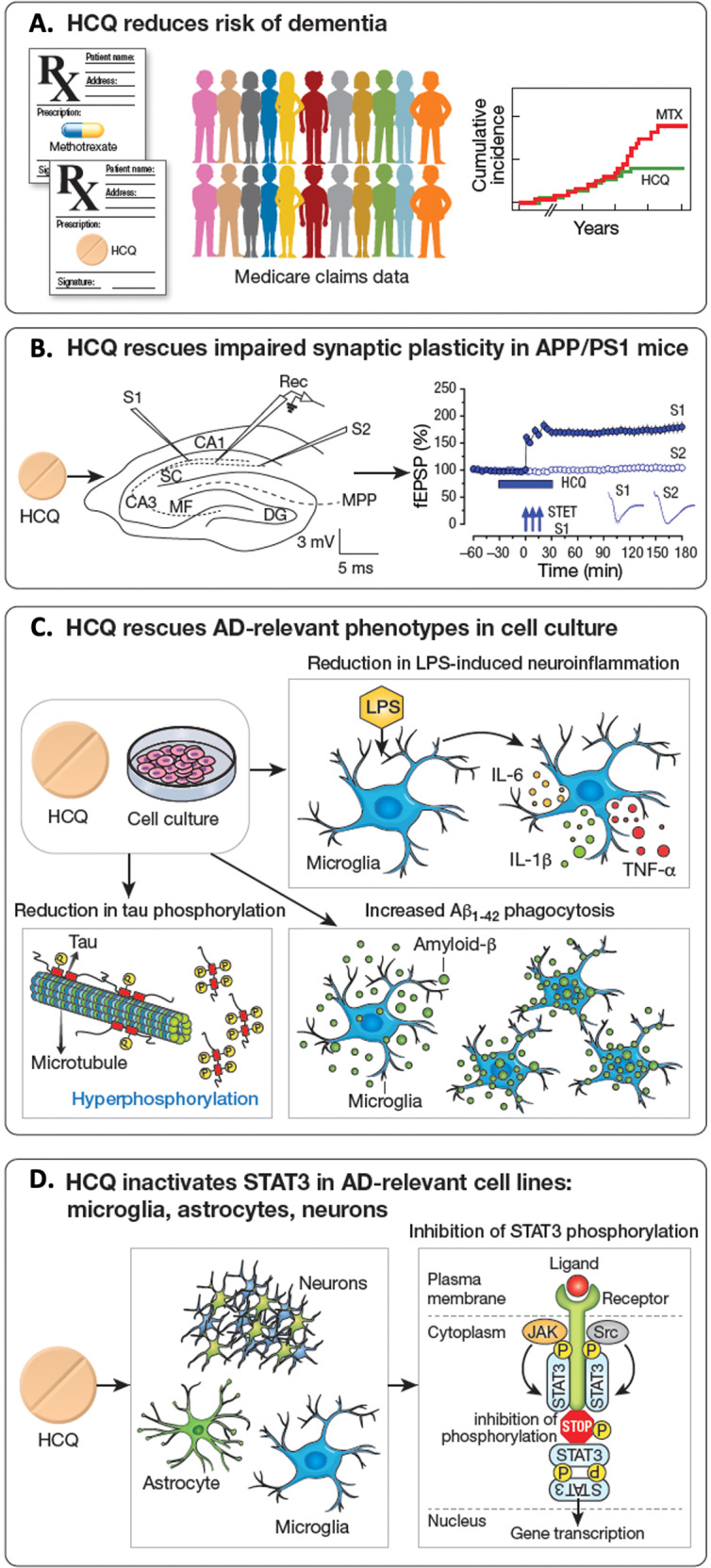
Study design and key findings. **A** We first demonstrated that in a large, real-world clinical dataset using Medicare claims data, exposure to HCQ reduces risk of incident ADRD in RA patients relative to the active comparator, methotrexate (MTX). **B** We next showed that HCQ rescues impaired hippocampal synaptic plasticity assessed by late long-term potentiation (LTP) in the APP/PS1 transgenic mouse model of AD. **C** We demonstrated that HCQ rescues molecular abnormalities associated with AD including reduction in LPS-induced neuroinflammation, increase in Aβ_1-42_ phagocytosis by microglia; and lowering of tau phosphorylation. **D** We finally demonstrated that HCQ inactivates STAT3 in microglia, astrocytes, and neurons. HCQ hydroxychloroquine, AD Alzheimer’s disease, LPS bacterial lipopolysaccharide, IL-1β interleukin 1 beta, TNF-α tumor necrosis factor alpha, Aβ_1-42_ amyloid-beta 1-42, APP/PS1 double transgenic mice expressing mutant human amyloid precursor protein and mutant human presenilin 1, SC Schaffer collateral pathway, CA1 cornu ammonis 1, CA3 cornu ammonis 3, MF mossy fiber, Rec recording electrode, S1 apical dendritic input, S2 basal dendritic input, MPP medial perforant path, fEPSP field excitatory postsynaptic potentials, STET strong tetanization, ADRD Alzheimer’s disease and related dementias, MTX methotrexate, RA rheumatoid arthritis.

In this study (Fig. 1) we first used a large, real-world clinical dataset to assess whether exposure to HCQ in older individuals lowers risk of incident Alzheimer’s disease and related dementias (ADRD). We then tested whether HCQ can restore impaired hippocampal synaptic plasticity in the APP/PS1 transgenic mouse model of AD and whether HCQ rescues molecular abnormalities associated with AD pathogenesis in cell culture-based phenotypic assays. Finally, we tested whether HCQ inactivates STAT3 in microglia, astrocytes, and neurons and whether restoration of impaired hippocampal synaptic plasticity is associated with STAT3 inactivation. Together, our results suggest that HCQ targets multiple molecular abnormalities in AD and may be a novel disease-modifying treatment in individuals in preclinical stages of AD progression. These findings merit confirmation in adequately powered human clinical trials.

## Materials and methods

### HCQ and clinically diagnosed incident ADRD in medicare claims data analyses

The full study protocol for patient-level analysis in Medicare claims was pre-registered on clinicaltrials.gov prior to data analysis (NCT04691505) and contains detailed information on implementation including all codes that were used to identify study variables to allow future replication. These analyses were performed within the ongoing DREAM study [3]. The following sections summarize key methodologic details.

#### Data source

We used Medicare Fee-For-Service claims data from 2007 through 2017. Medicare Part A (hospitalizations), B (medical services), and D (prescription medications) claims are available for research purposes through the Centers for Medicare and Medicaid Services (CMS). A signed data use agreement with the CMS was available and the Brigham and Women’s Hospital’s Institutional Review Board approved this study. All the analyses were conducted using anonymized patient data, therefore the institutional review board waived the requirement for informed consent.

#### Study cohort

We employed a new user, active comparator, observational cohort study design comparing HCQ with methotrexate (MTX). We selected MTX as a comparator to HCQ because both treatments are used first-line for RA. The patients were required to have continuous enrollment in Medicare parts A, B, and D during the baseline period of 365 days before initiation date of MTX or HCQ, which was defined as the cohort entry date. Patients were required to have ≥1 diagnosis codes indicating rheumatoid arthritis during the baseline period but no prior use of any disease modifying antirheumatic treatments. We excluded patients with existing diagnoses of ADRD any time prior to and including cohort entry date to focus on incident events. We further excluded patients with nursing home admission in the 365 days prior to and including cohort entry date as medication records for short nursing home stays are unavailable in Medicare claims.

#### Outcome measurement

We identified the endpoint of ADRD based on diagnosis codes, recorded on 1 inpatient claim or 2 outpatient claims indicating Alzheimer’s disease, vascular dementia, senile, presenile, or unspecified dementia, or dementia in other diseases classified elsewhere (see Supplementary Table 1). When validated against a structured in-home dementia assessment, Medicare claims-based dementia identification is reported to have a positive predictive value (PPV) in the range of 65% to 78% [12].

#### Alternative analytic approaches

To accommodate various uncertainties involved in pharmacoepidemiologic investigations focused on ADRD risk, we employed the following alternative analyses (Supplementary Fig. 1) recommended as good practice [13] and detailed earlier [3].

##### Analysis 1- ‘As-treated’ follow-up approach

In this approach, the follow-up started on the day following the cohort entry date and continued until treatment discontinuation or switch (to comparator treatment), insurance disenrollment, death, or administrative endpoint (December 2017). A 90-day ‘grace period’ after the end of the expected days-supply of the most recently filled prescription was considered to define the treatment discontinuation date to accommodate for suboptimal adherence during treatment periods.

##### Analysis 2- ‘As-started’ follow-up approach incorporating a 6-month ‘induction’ period

In this approach, we incorporated a 6-month induction period after the cohort entry date before beginning the follow-up for ADRD and followed patients for a maximum of 3 years regardless of subsequent treatment changes or discontinuation, similar to an intent-to-treat approach in randomized controlled trials. This follow-up approach addresses concerns related to informative censoring if patients discontinue or if physicians de-prescribe the treatments under consideration because of memory problems associated with ADRD, but the diagnosis is not recorded in the electronic healthcare records until after the drug is discontinued.

##### Analysis 3- Incorporating a 6-month ‘symptoms to diagnosis’ period

In this approach, we assigned an outcome date that is 6 months before the first recorded ADRD date and excluded last 6 months of follow-up for those who are censored without an event to account for the possibility that ADRD symptoms likely appear some time before a formal diagnosis is recorded in insurance records, which leads to misclassification of ADRD onset.

##### Analysis 4- Alternate outcome definition

In this approach, the outcome was defined using a combination of a diagnosis code and ≥1 prescription claim for a symptomatic treatment [donepezil, galantamine, rivastigmine, and memantine] occurring within 6 months of each other with outcome date assigned to second event in the sequence. Use of medication records to identify dementia has been reported to result in >95% PPV in a previous validation study [14].

#### Pre-treatment patient characteristics

We identified 73 patient characteristics, measured during the 365-days before the cohort entry date. The following set of variables were included: (1) demographic factors such as age, gender, race, socioeconomic status proxies, (2) risk factors for ADRD identified in previous studies such as diabetes, stroke, and depression [15–17], (3) lifestyle factors such as smoking as well as use of preventive services, including screening mammography and vaccinations, to account for healthy-user effects [18]; measures for use of various healthcare services before cohort entry including number of distinct prescriptions filled, number of emergency department visits, hospitalizations, and number of physician office visits to account for patients’ general health and contact with the healthcare system to minimize the possibility of differential surveillance [19]; frailty indicators based on composite scoring scheme [20] to address potential confounding by frailty, and (4) comorbid conditions and co-medications including prior use of steroids and opioids. Please refer to Supplementary Table 2 for a full list of covariates included.

#### Statistical analyses

We used a propensity-score (PS) [21]-based approach to minimize confounding in this study. The PS was calculated as the predicted probability of initiating the exposure of interest (HCQ) versus the reference drug (MTX) conditional on baseline covariates using multivariable logistic regression. On average, patients with similar PSs have similar distribution of potential confounders used to estimate the PS. Therefore, analyses conditioned on the PS provide effect estimates that account for confounding by these measured characteristics. For all our analyses, initiators of the exposure of interest (HCQ) were matched with initiators of the reference exposure (MTX) based on their PS [22]. Pair matching was conducted using a nearest-neighbor algorithm, which seeks to minimize the distance between propensity scores in each pair of treated and reference patients [23], and a caliper of 0.025 on the natural scale of the PS was used to ensure similarity between the matched patients [24]. Multiple diagnostics for PS analysis were evaluated including PS distributional overlap before and after matching to ensure comparability of these groups [25] and balance in each individual covariate between two treatment groups using standardized differences [26].

In the PS-matched population, incidence rates along with 95% confidence intervals for ADRD were estimated for the HCQ and MTX groups. The competing risk of death could have been of concern for the current set of analyses if mortality was frequent among patients included in the cohort and if differences in the risk of death between treatment and reference groups were substantial. Therefore, we calculated cumulative incidence of ADRD using cumulative incidence functions that account for competing risk by death and provided cause-specific hazard ratios from Cox proportional hazards regression model [27]. We used cumulative incidence functions that estimate the probability of experiencing ADRD at each time point considering death as a competing event and separately estimating survival probability. Further, Cox proportional hazards regression model was used to estimate cause-specific hazard ratios for ADRD accounting for competing risk of death by censoring on all-cause mortality. The cumulative incidence plots were inspected visually for evidence of violation of the non-proportionality assumption; gross violations were not identified. Pre-specified subgroup analyses were conducted based on age, sex, and baseline cardiovascular disease.

We conducted two secondary analyses: first we restricted the outcome definition to only include codes for AD. Second, we switched the reference drug from MTX to leflunomide, the third most commonly used non biologic DMARD in Medicare after MTX and HCQ. These secondary analyses were used to evaluate the consistency of any effects in the primary analyses.

Assuming an incidence rate of incident AD dementia of 1.23 per 100 person-years as reported previously based on the Baltimore Longitudinal Study of Aging [28], we estimated that a total of 9625 patients treated with candidate treatment and 9625 matched patients treated with reference medication will be needed to detect 20% reduction in the incidence rate of dementia over 3 years with 80% power. Our study sample exceeded this estimated sample size requirement.

Analyses of the Medicare claims data were performed using the Aetion Evidence Platform v4.32 (incl. R v3.4.2), which has been scientifically validated by accurately repeating a range of previously-published studies [29] and by replicating [30] or predicting clinical trial findings [31].

### HCQ and hippocampal synaptic plasticity in APP/PS1 mice

#### Animals

All animal experiments and procedures were approved by the Institutional Animal Care and Use Committee (IACUC) of National University of Singapore. We used a transgenic mouse model of AD, which expresses a mutated chimeric mouse/human APP and the exon-9-deleted variant of human PS1, both linked to familial AD, under the control of a prion promoter element (*APPSwe/PS1dE9*), which we denote as APP/PS1 [32, 33]. A total of 35 hippocampal slices (21 APP/PS1 and 14 wild type (WT) slices) were prepared from 9 APP/PS1 mice and 7 WT mice which were 4–5 months old. Animals were housed under 12 h light/12 h dark conditions with food and water available ad libitum. Power calculations were not carried out for APP/PS1 hippocampal synaptic plasticity experiments. We followed standard procedures for our vitro slice physiology for long-term functional plasticity studies. This includes a sample size of 7/9 slices as well as nonparametric testing of differences. These are similar sample sizes used in prior LTP experiments from Sajikumar et al. Animals were not randomized to experimental groups and investigators were not blinded to allocation.

#### Hippocampal slice preparation

Animals were anaesthetized briefly using CO_2_ and were decapitated. Brains were quickly removed in 4 °C artificial cerebrospinal fluid (aCSF), a modified Krebs-Ringer solution containing the following (in mM): 124 NaCl, 3.7 KCl, 1.2 KH_2_PO_4_, 1 MgSO_4_·7H_2_O, 2.5 CaCl_2_·2H_2_O, 24.6 NaHCO_3_, and 10 d-glucose. The pH of aCSF was between 7.3 and 7.4 when bubbled with 95% oxygen and 5% carbon dioxide (carbogen). Both right and left hippocampi were dissected out in cold (2–4 °C) aCSF, which was continuously bubbled with carbogen [34–36]. Transverse hippocampal slices of 400 μm thickness were prepared from the right and left hippocampus using a manual tissue chopper (Stoelting, Wood Dale, Illinois), transferred onto a nylon net placed in an interface chamber (Scientific Systems Design, Ontario, Canada) and incubated at 32 °C at an aCSF flow rate of 1 ml/min and carbogen consumption of 16 l/h. The entire process of animal dissection, hippocampal slice preparation and placement of slices on the chamber was done within approximately five minutes to ensure that hippocampal slices were in good condition for electrophysiology studies. The slices were incubated for at least 3 h before starting the experiments. Detailed description of these methods has been reported previously [35, 36].

#### Field potential recordings

In all the electrophysiology recordings, two-pathway experiments were performed. Two monopolar lacquer-coated stainless-steel electrodes (5MΩ; AM Systems, Sequim) were positioned at an adequate distance within the stratum radiatum of the CA1 region for stimulating two independent synaptic inputs S1 and S2 of one neuronal population, thus evoking field excitatory postsynaptic potentials (fEPSP) from Schaffer collateral/commissural‐CA1 synapses. One electrode (5MΩ; AM Systems) represented as ‘rec’ was placed in the CA1 apical dendritic layer for recording fEPSP. After the pre‐incubation period, a synaptic input-output curve (afferent stimulation vs. fEPSP slope) was generated. Test stimulation intensity was adjusted to elicit fEPSP slope of 40% of the maximal slope response for both synaptic inputs S1 and S2. The signals were amplified by a differential amplifier, digitized using a CED 1401 analog-to-digital converter (Cambridge Electronic Design, Cambridge, UK) and monitored online with custom-made software. To induce late long-term potentiation (LTP), a “strong” tetanization (STET) protocol consisting of three trains of 100 pulses at 100 Hz (single burst, stimulus duration of 0.2 ms per polarity), with an inter‐train interval of 10 min, was used. In all experiments, a stable baseline was recorded for at least 30 min. Four 0.2-Hz biphasic, constant current pulses (spaced at 5 s) given every five min were used for baseline and post-induction recordings also and the average slope values from the four sweeps was considered as one repeat while used for plotting. Initial slopes of fEPSPs were expressed as percentages of baseline averages. A series of pulses ranging from 0, 10, 20, 30, 40, 50, 70, 100 microamperes were applied to generate an input-output curve. Graphs are plotted as stimulus intensity versus fEPSP slope. Paired pulse ratio (PPR) was evoked using an interstimulus interval of 50 ms at 40 % of maximum stimulus intensities. PPR was expressed as the ratio of the fEPSP slope of second stimulus to the first stimulus [37].

#### Pharmacology

HCQ Sulphate (Selleckchem, catalog, No-S4430) was stored at −20 °C as 50 mM stock in deionized water. Before application, the stock solution was diluted to a final concentration of 25 µM or 50 µM in aCSF and bath-applied for a total of 60 min, 30 min before and 30 min after the STET or unless otherwise specified.

MTX was stored similarly and diluted to a final concentration of 50 nM in aCSF and bath-applied for a total of 60 min, 30 min before and 30 min after the STET or unless otherwise specified.

#### Statistical analysis

All data are represented as mean ± standard error of the mean (SEM). The fEPSP slope value expressed as percentages of average baseline values per time point was subjected to statistical analysis using Graph Pad Prism (Graph Pad, San Diego, CA, USA). Nonparametric tests were used considering lack of normality due to small sample size. Wilcoxon signed rank test (Wilcox test) was used to compare fEPSP values within one group and Mann-Whitney U test (U-test) was used when data were compared between groups. Statistical comparisons for input-output (I/O) curve and paired pulse facilitation (PPF) experiments were performed using two-way ANOVA test. *p* < 0.05 was considered as the cutoff for statistically significant differences.

### HCQ and AD-related phenotypes in cell culture

We tested whether HCQ could rescue molecular phenotypes relevant to AD including Aβ_1-42_ clearance, Aβ secretion, Aβ toxicity, tau phosphorylation, lipopolysaccharide (LPS)-induced neuroinflammation, cell death due to trophic factor withdrawal and neurite outgrowth. LPS-induced neuroinflammation assays were performed on BV-2 cells (immortalized murine microglial cells) and adult 5xFAD microglia. Aβ_1-42_ clearance studies were performed on BV-2 cells, adult 5xFAD microglia, and iPSC derived microglia from an adult human AD patient. Aβ secretion assay was performed on human APP overexpressing H4-hAPP cells, Aβ toxicity studies were performed on primary hippocampal neurons, tau phosphorylation in tau441 overexpressing SH-SY5Y cells, and cell death due to trophic factor withdrawal and neurite outgrowth on mouse primary cortical neurons. Supplementary Table 3 includes descriptions of the phenotypic assays and HCQ concentrations tested. Detailed descriptions of phenotypic assays are included in Supplementary Text. Cell lines used for phenotypic assays are regularly tested for mycoplasma contamination by PCR of cell culture supernatants. Only mycoplasma free cells were used for experiments. All experiments were conducted on cells that have been passaged no more than 5 times upon thawing. Transgenic cell lines are regularly assessed for transgene expression on protein level.

#### Statistical analyses

Statistical analysis was performed in GraphPad Prism 9.1.2. Group differences were evaluated for each test item separately by one-way ANOVA followed by Dunnett’s multiple comparison test versus VC or lesion control.

### HCQ and STAT3 inactivation in microglia, astrocytes, and neurons

Immortalized human microglia HMC3 cells (ATCC CRL − 3304) and astrocytoma 1321N1 cells (Sigma-Aldrich; derived from human brain astrocytoma; [38]) were cultured in Eagle’s minimum essential medium (EMEM) supplemented with 10% fetal bovine serum (FBS, Gibco) and 1% antibiotics and antimycotics (Gibco). Neuroblastoma SK-N-BE(2)-M17 (M17) (ATCC CRL-2267) cells were cultured in a 1:1 mixture of EMEM and F12 media, supplemented with 10% FBS plus 1% antibiotics and antimycotics.

For primary cultures of embryonic cortical neurons, timed-pregnant mice were obtained from the Jackson Laboratory. Cultures were prepared from embryonic day E18.5 cerebral tissues as described previously [39]. Pregnant mice were killed by fast cervical dislocation, embryos and embryo brains were removed, and the cerebral hemisphere was extracted in sterile Hank’s balanced saline solution (HBSS). Brain tissues were incubated in 0.25% trypsin-EDTA for 30 min at 37 °C and then transferred to Dulbecco’s Modified Eagle Medium (DMEM) containing 10% fetal bovine serum (DMEM+). The tissues were transferred to neurobasal (NB) medium containing B27 supplements, 2 mM L-glutamine, antibiotics and antimycotics (Gibco), and 1 mM HEPES and dissociated by trituration using a fire-polished Pasteur pipet. The dissociated cells were seeded into polyethyleneimine-coated plastic culture dishes at a density of 60,000 cells/cm^2^ and cultured in the same B27-containing NB medium. Experiments were performed 5 days later.

Treatment with HCQ Sulfate (Selleckchem, Catalog No.S4430) for 48 h was performed at a concentration of 50 µM. Treatment with Methotrexate (Selleckchem, Catalog No.S5097) for 48 h was performed at a concentration of 50 nM. Both treatment doses were previously assessed to confirm that they did not produce adverse effects on cell viability assessed by direct cell counting.

#### Western blot analyses

Protein extracts were obtained by lysing cells with a denaturing buffer containing 2% sodium dodecyl sulfate (SDS) (Sigma-Aldrich) in 50 mM HEPES. After boiling and sonication, whole-cell protein extracts were size-fractionated through polyacrylamide gels and transferred to nitrocellulose membranes (Bio-Rad). Membranes were blocked with 5% non-fat dry milk and immunoblotted. Primary antibodies were employed that recognized total STAT3 (124H6) (Cell Signaling, 9139 T) and phosphorylated STAT3 (p-STAT3) (Tyr705) (D3A7) XP® (Cell Signaling, 9145 S), the primary phosphorylated STAT3 residue.

#### Statistical analyses

Digitized images were obtained, processed, and quantified with ImageLab version 6.1 (BioRad Laboratories) and densitometry data was analyzed with ImageJ. We tested β-Actin (loading control)-normalized total STAT3, p-STAT3 and the ratio of p-STAT3/total STAT3 in HCQ-treated and MTX-treated cells compared to untreated control cells using the one-way ANOVA test (parametric). We additionally used the Wilcoxon rank-sum test (non-parametric) to confirm that results were robust to distributional assumptions. Significant differences were indicated as *p* < 0.05.

### HCQ and hippocampal synaptic plasticity in APP/PS1 mice, and inactivation of STAT3

#### Western blot analyses

For Western blot analyses, we studied four groups (3 mice in each group, 5 months old) in two experiments (HCQ at 25 or 50 µM): (i) WT, (ii) APP/PS1, (iii) WT with HCQ 25 or 50 µM and (iv) APP/PS1 with HCQ 25 or 50 µM. Hippocampal slices were treated with drug 30 min before and after STET. In each group, the slices were collected one hour after STET. Tissues around the recording electrodes in CA1 region were cut carefully and snap frozen in liquid nitrogen and stored at −80 °C. Protein extraction was performed using Tissue Protein Extraction reagent (T-PER; Thermo Scientific, USA) supplemented with protease and phosphatase inhibitor (Thermo Scientific, USA) according to the manufacturer’s protocol, followed by centrifugation for 5 min at 10,000 rpm at 4 °C. Protein concentration was determined using the Bradford assay (Bio-Rad). Appropriate protein concentration was added to the sample buffer and heated at 95 °C for 10 min before separation on SDS-polyacrylamide gels. Gels were transferred to PVDF membranes (Bio-Rad) in a wet transfer cell (Bio-Rad) for 1.5 h at 100 V. The membranes were blocked with 5% w/v non-fat dry milk in 1X TBST and immunoblotted with primary antibodies. The primary antibodies with their concentrations used were as follows: rabbit anti-phospho STAT3 (Tyr705) (1:1000, Cell Signaling), mouse anti-STAT3 (1:2000, Cell Signaling) and mouse anti-tubulin (1:20000; Sigma-Aldrich). The membranes were incubated with secondary peroxidase-conjugated antibodies. Signals were generated by using the SuperSignal® West Pico Chemiluminescent Substrate (Thermo Scientific, USA).

#### Statistical analyses

Digitized images were obtained, processed, and quantified using ImageJ (NIH software). We examined tubulin (loading control)-normalized total STAT3, p-STAT3 and the ratio of p-STAT3/total STAT3 in APP/PS1 and WT, HCQ treated and untreated hippocampal slices. All values were calculated in relation to the control group (i.e., WT). Similar to western blot analytic methods described above, group differences comparing HCQ-treated cells to untreated control cells were evaluated using the one-way ANOVA test (parametric). We additionally used the Wilcoxon rank-sum test (non-parametric) to confirm that results were robust to distributional assumptions. Significant differences were indicated as *p* < 0.05.

## Results

### HCQ lowers risk of clinically diagnosed incident ADRD compared to MTX in medicare claims data

To test whether exposure to HCQ lowers ADRD risk in older individuals, we used longitudinal insurance claims data from Medicare beneficiaries (NCT04691505). We assembled a cohort of patients with rheumatoid arthritis (RA), an indication for which HCQ is routinely used, to identify initiators of HCQ or an active comparator, methotrexate (MTX). After controlling for 73 confounding variables through PS matching, we estimated treatment effects in four alternative analyses designed to address various uncertainties associated with claims-based analyses of dementia risk including exposed person-time misclassification, reverse causation, informative censoring, and misclassification of outcome onset as described previously (Supplementary Fig. 1; DREAM study) [3].

Of the 881,432 patients filling at least one prescription for the drugs of interest (HCQ or MTX) during our study period, we included 109,124 patients with RA who met all inclusion criteria (54,562 HCQ initiators 1:1 PS matched to 54,562 MTX initiators). Supplementary Table 2 provides patient characteristics before and after PS matching. As HCQ and MTX are both used as first-line DMARDs for RA, we noted that most characteristics were balanced even before PS matching indicating sufficient clinical equipoise and comparability of the two exposure groups. Average age of included patients was 74 years, 76% were females, and 84% were White. PS-matching minimized all residual imbalances in measured characteristics.

**Fig. 2:**
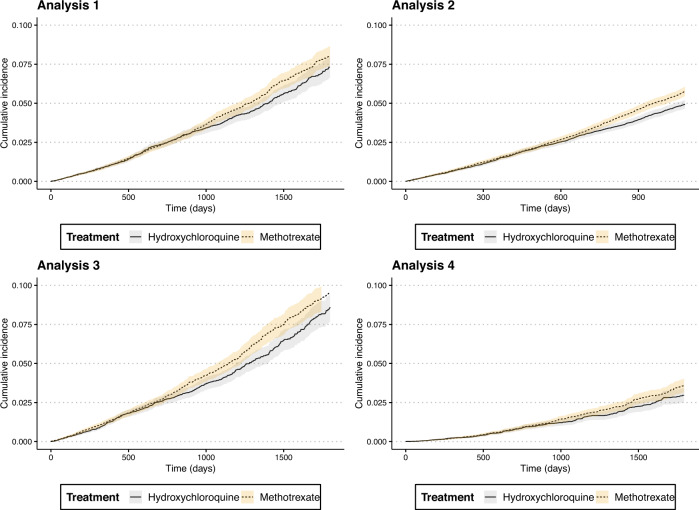
Cumulative incidence of clinically diagnosed Alzheimer’s and related dementia (ADRD) in rheumatoid arthritis patients treated with methotrexate or hydroxychloroquine, medicare data 2007–2017. A new-user active comparator design with propensity score (PS)-based adjustment for confounding, was used to estimate treatment effects in four alternative analyses. Analyses indicate that cumulative incidence of ADRD among HCQ initiators compared to MTX initiators diverged after approximately 2 years of treatment wherein individuals on HCQ had lower cumulative incidence of ADRD compared to MTX users. The four analyses were designed to address various uncertainties associated with claims-based analyses of ADRD risk: Analysis 1: ‘As-treated’ follow-up approach (MTX: *N* patients = 54,562, *N* outcomes = 1096, *N* person years = 71,029; HCQ: *N* patients = 54,562, *N* outcomes = 774, *N* person years = 56,891); Analysis 2: ‘As-started’ follow-up approach incorporating a 6-month induction period (MTX: *N* patients = 30,615, *N* outcomes = 1391, *N* person years = 68,504; HCQ: *N* patients = 30,615, *N* outcomes = 1206, *N* person years = 68,329); Analysis 3: Incorporating a 6-month ‘symptom to diagnosis’ period’ period (MTX: *N* patients = 25,072, *N* outcomes = 798, *N* person years = 43,254; HCQ: *N* patients = 25,072, *N* outcomes = 609, *N* person years = 39,808); and Analysis 4: Alternate outcome definition (MTX: *N* patients = 54,562, *N* outcomes = 416, *N* person years = 71,669; HCQ: *N* patients = 54,562, *N* outcomes = 275, *N* person years = 57,349). See Methods for additional description of analytic approach. HCQ hydroxychloroquine, MTX methotrexate.

**Fig. 3:**
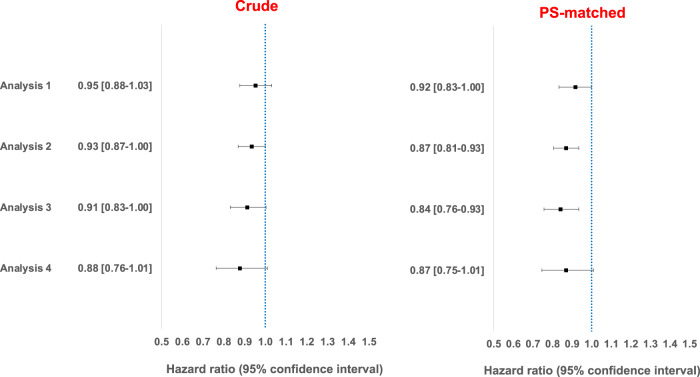
Comparative risk of clinically diagnosed Alzheimer’s and related dementia (ADRD) in rheumatoid arthritis patients treated with hydroxychloroquine versus methotrexate, medicare data 2007–2017. A new-user active comparator design with PS-based adjustment for confounding, was used to estimate treatment effects in four alternative analyses. Analyses indicate that the that risk of ADRD was consistently lower among HCQ initiators. The four analyses were designed to address various uncertainties associated with claims-based analyses of ADRD risk: Analysis 1: ‘As-treated’ follow-up approach; Analysis 2: ‘As-started’ follow-up approach incorporating a 6-month induction period; Analysis 3: Incorporating a 6-month ‘symptom to diagnosis’ period’ and Analysis 4: Alternate outcome definition (See Methods for additional description of analytic approach). HCQ hydroxychloroquine, MTX methotrexate, PS propensity score.

Supplementary Table 4 summarizes the incidence rates of clinically diagnosed incident ADRD per 1000 person years (95% confidence intervals [CI]) in the two exposure groups across the four analyses showing lower incidence rates for ADRD among individuals exposed to HCQ compared to MTX. Figure 2 summarizes cumulative incidence of ADRD among HCQ initiators compared to MTX initiators after accounting for competing risk by mortality; results from all four analyses indicate that after approximately 2 years of treatment, individuals on HCQ had lower cumulative incidence of ADRD compared to MTX. Figure 3 summarizes the crude and PS-matched comparative risk of ADRD in HCQ compared to MTX groups; results indicated that risk of ADRD was consistently lower among HCQ initiators. In Analysis 1 where patient follow-up time was censored at discontinuation of the initial treatment (“as-treated” approach), HCQ initiators had an 8% lower rate of ADRD compared to MTX initiators (HR 0.92 [95% CI 0.83–1.00]). In Analysis 2 where we incorporated a 6-month induction period to eliminate potential reverse causality and continued follow-up for 3 years regardless of treatment change or discontinuation to minimize the impact of informative censoring in an ‘as started’ follow-up scheme, HCQ was significantly associated with a 13% lower rate (HR 0.87 [95% CI 0.81–0.93]). In Analysis 3 where we accommodated a 6-month ‘symptom to claims’ period to address misclassification of outcome onset, HCQ was significantly associated with a 16% lower rate (HR 0.84 [95% CI 0.76–0.93]). Finally, in Analysis 4, which required symptomatic treatment with cholinesterase inhibitors or memantine along with diagnosis codes to overcome outcome misclassification due to lower specificity of the outcome measurement relying only on diagnosis codes, effect estimates were largely consistent with other approaches (HR 0.87 [95% CI 0.75–1.01]). We observed no conclusive evidence of heterogeneity in treatment effects across subgroups of age, gender, and baseline cardiovascular function (Supplementary Fig. 2).

In secondary analyses, restricting the outcome to AD only, we observed similar results comparing HCQ to MTX (Analysis 1: as treated: HR 0.78 [95% CI 0.65–0.93]). Using leflunomide as the active comparator in place of MTX, we also observed similar results (Analysis 1: HCQ vs leflunomide: as treated: HR 0.76 [95% CI 0.65–0.88]).

### HCQ rescues impaired hippocampal synaptic plasticity in APP/PS1 mice

To test whether HCQ may rescue impaired hippocampal synaptic plasticity in AD, we studied its effects on late long-term potentiation (LTP), a form of activity-induced synaptic plasticity that has been shown to be impaired prior to significant accumulation of amyloid plaques and neurodegeneration in the APP/PS1 transgenic AD mouse model [33].

**Fig. 4:**
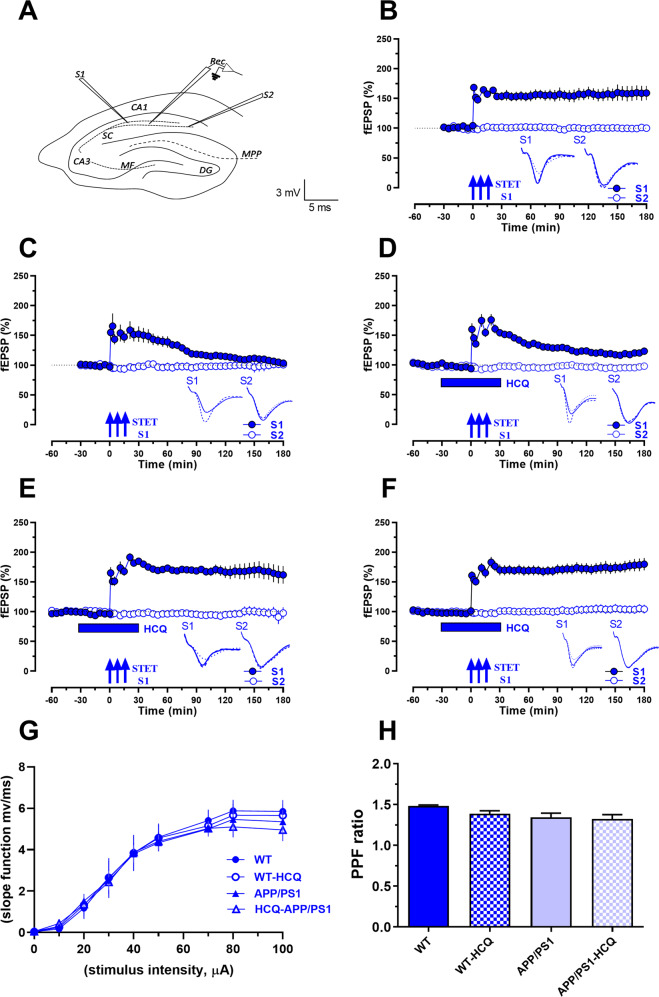
Treatment with HCQ rescues late LTP in hippocampal CA1 synapses of APP/PS1 mice. **A** Schematic representation of a hippocampal slice with electrodes located in the CA1 region. ‘Rec’ represents the recording electrode positioned in the CA1 region flanked by two stimulating electrodes represented as S1 and S2 in the stratum radiatum to stimulate two independent pathways to a single neuronal population in the Schaffer collateral pathway (sc). **B** Induction of late LTP by STET in synaptic input S1 in WT mice resulted in a potentiation that remained stable for 180 min (filled blue circles, *n* = 7). **C** Induction of late LTP by STET in synaptic input S1 in APP/PS1 mice resulted only in early LTP in S1 (filled blue circles, *n* = 6). **D** Treatment of hippocampal slices with 25 µM HCQ resulted in late LTP in S1 in APP/PS1 mice (filled blue circles, *n* = 8) that was however significantly lower in magnitude than WT late LTP (D vs B). **E** Treatment of hippocampal slices with 50 µM HCQ resulted in late LTP in S1 in APP/PS1 mice (filled blue circles, *n* = 7) similar to WT late LTP (**E** vs **B**). **F** Treatment of hippocampal slices with 50 µM HCQ in WT mice resulted in late LTP in S1 that was similar to untreated WT late LTP (**F** vs **B**). In Figs. (**B**–**F**), control input S2 remained stable throughout the recording (open blue circles). **G** Comparison of input-output curves showed no significant change between WT and APP/PS1 before and after HCQ application. **H** Comparison of PPR also revealed no significant change in PPF ratio between WT and APP/PS1 mice before and after HCQ application (*n* = 12). Error bars in all the graphs indicate ±SEM. Analog traces represent typical fEPSPs of inputs S1 and S2, recorded 15 min before (dotted line), 30 min after (dashed line), and 180 min (solid line) after tetanization in S1 and the corresponding time points in S2. The three solid arrows represent the time of induction of late LTP by STET. Blue rectangular bar represents the time of application of HCQ. Scale bars: vertical, 2 mV; horizontal, 3 ms. HCQ hydroxychloroquine, PPR paired pulse ratio, APP/PS1 double transgenic mice expressing AD pathology (human amyloid precursor protein mutant human presenilin 1), WT wild type mice, LTP long-term potentiation, STET strong tetanization, PPF paired pulse facilitation, SEM standard error of the mean, fEPSP field excitatory postsynaptic potential.

Figure 4A illustrates the schematic diagram of the location of electrodes in a hippocampal slice from wild type (WT) and APP/PS1 transgenic mice (ages 4–5 months). Figures 4B, C illustrate late LTP recordings from the hippocampus of non-disease/WT controls and disease (APP/PS1) mice respectively. In WT hippocampal slices, applying strong tetanization (STET; S1 input) resulted in a long-lasting and stable late LTP throughout the recording time period of 180 min (Fig. 4B: filled blue circles). The control input (S2) remained stable throughout the recording time period (Fig. 4B: open blue circles). We observed statistically significant differences in field excitatory postsynaptic potentials (fEPSP) from 1 min until 180 min when compared with its own baseline and with control input S2 (1 min Wilcox, *p* = 0.01, U-test, *p* = 0.0006; 60 min Wilcox, *p* = 0.01, U-test, *p* = 0.0006; 120 min Wilcox, *p* = 0.01, U-test, *p* = 0.0006; 180 min Wilcox, *p* = 0.01, U-test, *p* = 0.0006 respectively).

In APP/PS1 hippocampal slices, induction of late LTP by applying STET to S1 resulted in only short-lasting form of LTP (early-LTP) (Fig. 4C: filled blue circles) while the control input S2 remained stable (Fig. 4C: open blue circles). Significant difference was observed in fEPSP only until 170 min, when compared to its own baseline and until 115 min when compared to S2 (170 min Wilcox, *p* = 0.03, 115 min U-test, *p* = 0.04 respectively).

We then tested whether HCQ (25 µM) could rescue impaired late LTP in hippocampal slices from APP/PS1 mice. As shown in Fig. 4D, bath-application of HCQ 30 min before and 30 min after the STET induced late LTP in the APP/PS1 hippocampus (Fig. 4D: filled blue circles) which was significantly different from 1 min up to 180 min when compared to its own baseline and S2 (Fig. 4D: open blue circles) (1 min Wilcox, *p* = 0.007, U-test, *p* = 0.0002; 60 min Wilcox, *p* = 0.007, U-test, *p* = 0.0002; 120 min Wilcox, *p* = 0.007, U-test, *p* = 0.0003; 180 min Wilcox, *p* = 0.01, U-test, *p* = 0.01 respectively). When compared to WT (Fig. 4D vs 4B), late LTP in HCQ (25 µM) -treated APP/PS1 group was similar in magnitude up to 60 min (U-test, *p* > 0.05). From 70 min through 180 min, late LTP in APP/PS1 hippocampus after HCQ application (25 µM) showed that potentiation remained significantly lower than in WT (70 min, U-test, *p* = 0.009; 180 min, U-test, *p* = 0.01) indicating a partial rescue of impaired late LTP at this dose (Fig. 4D vs 4B).

To assess whether HCQ exerted dose-dependent effects on late LTP in APP/PS1 mice, we next tested a higher concentration of HCQ (50 µM) in these experiments. As shown in Fig. 4E, HCQ (50 µM) induced late LTP (S1) in the APP/PS1 hippocampus (Fig. 4E: filled blue circles). We observed statistically significant differences in fEPSP compared to its own baseline and S2 (Fig. 4E: open blue circles) up to 180 min (1 min Wilcox, *p* = 0.01, U-test, *p* = 0.0006; 60 min Wilcox, *p* = 0.01, U-test, *p* = 0.0006; 120 min Wilcox, *p* = 0.01, U-test, *p* = 0.0006; 180 min Wilcox, *p* = 0.01, U-test, *p* = 0.004 respectively). Late LTP in the HCQ (50 µM) treated APP/PS1 group was similar in magnitude to WT (U-test, *p* > 0.05 at 1, 60, 120, 180 min) (Fig. 4E vs 4B), throughout the recording period, indicating a complete rescue of late LTP at the higher dose of HCQ.

We also tested whether HCQ exerted any detrimental effects on hippocampal synaptic plasticity in WT mice. As shown in Fig. 4F, late LTP in HCQ-treated WT hippocampal slices (Fig. 4F: filled blue circles) was similar in magnitude compared to untreated WT (Fig. 4F vs 4B, U-test, *p* > 0.05). We observed statistically significant differences in fEPSP from 1 min until 180 min when compared with its own baseline (Wilcox, *p* = 0.01 at 1, 60, 120, 180 min) as well as with S2 (Fig. 4F: open blue circles) (U-test, *p* = 0.001 at 1, 60, 120, 180 min) throughout the recording period. In all experiments, control input S2 remained stable throughout the recording time period.

Comparison of input-output (I/O) curves between WT and APP/PS1 before and after HCQ application did not show any significant differences (*p* = 0.99) (Fig. 4G). Comparison of paired pulse ratio (PPR) in WT and APP/PS1 before and after HCQ did not show a significant difference (*p* = 0.09), suggesting that HCQ may not affect basal synaptic transmission in either WT or APP/PS1 mice (Fig. 4H).

Using the same experimental design, we additionally tested whether MTX (50 nM) affects impaired hippocampal synaptic plasticity in the APP/PS1 transgenic AD mouse model. Supplementary Fig. 3A indicates that MTX did not exert any detrimental effects on hippocampal synaptic plasticity in WT mice. Supplementary Fig. 3B indicates that MTX did not rescue impaired late LTP in hippocampal slices from APP/PS1 mice; there was no significant difference comparing MTX treated and untreated APP/PS1 hippocampal slices. See Supplementary Text for additional details.

### HCQ rescues AD phenotypes in cell culture

To test whether HCQ rescues molecular outcomes relevant to AD in cell culture-based phenotypic assays, we examined its effects on lipopolysaccharide (LPS)-induced neuroinflammation, Aβ_1-42_ clearance, Aβ_1-42_ toxicity, and Aβ secretion, tau phosphorylation, cell death due to trophic factor withdrawal, and neurite outgrowth and neurogenesis.

**Fig. 5:**
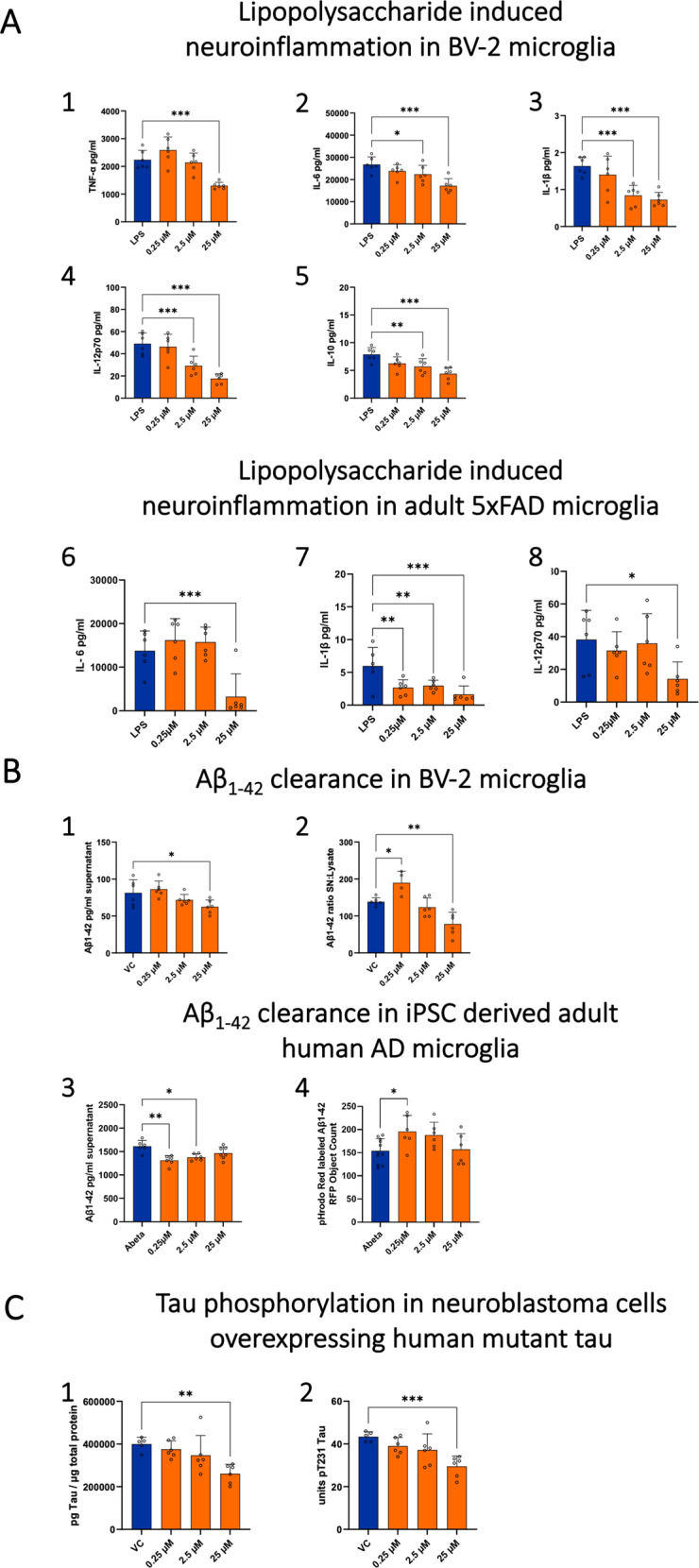
Hydroxychloroquine rescues molecular phenotypes relevant to AD. **A** Levels of inflammatory cytokines (1) TNFα, (2) IL-6, (3) IL-1β, (4) IL-12p70, and (5) IL-10 in the supernatant of BV-2 microglial cells and (6) IL-6, (7) IL-1β, (8) IL-12p70 in adult 5xFAD microglial cells after 24 h LPS stimulation and HCQ treatment. HCQ significantly reduced secretion of inflammatory cytokines in a dose-dependent manner. **B** Levels of Aβ_1-42_ in the (1) supernatant (2) supernatant:lysate Aβ_1-42_ in BV-2 microglial cells after 24 h treatment with HCQ and (3) supernatant and (4) uptake of pHrodo red positive cells into acidic cell organelles in iPSC derived adult human AD microglial cells after 4h treatment with HCQ. HCQ significantly increased clearance of Aβ_1-42_ as shown by reduced levels of Aβ_1-42_ in the supernatant and a lowering of the Aβ_1-42_ supernatant:lysate ratio and HCQ significantly increased microglial uptake of Aβ_1-42_. **C** Levels of (1) total tau and (2) phosphorylated tau (pT231), in lysates from SH-SY5Y cells over-expressing mutant human tau441 (SH-SY5Y-TMHT441) after 24 h treatment with HCQ. HCQ (25 µM) significantly reduced levels of total tau and phosphorylated tau (pT231). Error bars in all bar graphs indicate group mean + standard deviation (SD). Individual values are shown as dots (*n* = 6 per group). Group differences comparing HCQ-treated cells to the VC (**A** & **B**) or LPS control (**C**) were evaluated using the one-way ANOVA test followed by Dunnett’s multiple comparison test. Asterisks indicate significant differences between groups: *p < 0.05; **p < 0.01; ***p < 0.001. HCQ hydroxychloroquine, AU arbitrary units, VC vehicle control (0.1% DMSO), LPS lipopolysaccharide, iPSC induced pluripotent stem cells.

Treatment of BV-2 microglia with HCQ (2.5 µM, 25 µM) reduced the levels of secreted pro-inflammatory cytokines from microglia in the LPS-induced neuroinflammation assay. A reduction in TNF-α secretion was observed at the highest HCQ concentration (25 µM) and a dose-dependent reduction in IL-6, IL-1β, IL-12p70 and IL-10 was observed at 2.5 µM and 25 µM without any adverse effects on cell viability (Fig. 5A: 1–5).

Treatment of MACS isolated adult 5xFAD mouse microglia with HCQ reduced levels of IL-6, IL-12p70, and IL-10 at the highest HCQ concentration (25 µM) and reduced levels of IL-1β at all concentrations (0.25 µM, 2.5 µM, 25 µM) without any adverse effects on cell viability (Fig. 5A: 6–8)

**Fig. 6:**
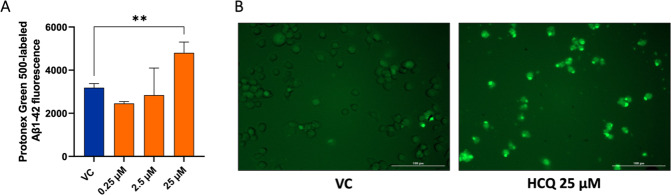
Hydroxychloroquine increases exogenous Aβ_1-42_ clearance through microglial uptake into acidic organelles. **A** HCQ (0.25, 2.5, and 25 µM) was applied to BV-2 microglial cells treated with Protonex Green 500-labelled Aβ_1-42_ (3 h). Labelled Aβ_1-42_ exhibits green fluorescence when internalized by acidic cell organelles (e.g., lysosomes) and is non-fluorescent at physiologic pH. BV-2 microglial cells treated with HCQ 25 µM showed significantly increased microglial uptake of Aβ_1-42_ into acidic cell organelles compared to control cells treated with Aβ_1-42_ alone. **B** Images of Protonex Green 500-labelled Aβ_1-42_ comparing the VC to HCQ 25 µM. Error bars in the bar graph indicate group mean + standard deviation (SD). Group differences comparing HCQ-treated cells to the VC were evaluated using the one-way ANOVA test. Asterisks indicate significant differences between groups: **p < 0.01 HCQ hydroxychloroquine, RFU relative fluorescence unit, VC vehicular control.

Treatment of BV-2 microglia with HCQ (25 µM) increased microglial clearance of exogenous Aβ_1-42_ as shown by reduced levels of Aβ_1-42_ in the supernatant and a lowering of the Aβ_1-42_ supernatant:lysate ratio (i.e. phagocytized Aβ_1-42_) without adverse effects on cell viability (Fig. 5B: 1, 2). Treatment with HCQ (25 µM) significantly increased microglial uptake of pH-sensitive Protonex-labelled Aβ_1-42_ into acidic organelles compared to control cells treated with Aβ_1-42_ alone (Fig. 6).

Treatment of iPSC derived adult human AD microglia with HCQ (0.25 µM, 2.5 µM) increased microglial clearance of exogenous Aβ_1-42_ as shown by reduced levels of Aβ_1-42_ in the supernatant (Fig. 5B: 3). Treatment with HCQ (0.25 µM) significantly increased microglia uptake of pHrodo red positive cells into acidic cell organelles compared to control cells treated with Aβ_1-42_ alone (Fig. 5B: 4)

Treatment of MACS isolated adult 5xFAD mouse microglia with HCQ did not have an effect on microglial clearance of exogenous Aβ_1-42_ at any concentration.

Treatment with HCQ (25 µM) reduced levels of total tau and phosphorylated tau in SH-SY5Y cells overexpressing human mutant tau (pT231) (Fig. 5C: 1,2).

A summary of results across all AD-related phenotypic assays is included in Supplementary Table 5.

### HCQ inactivates STAT3 in microglia, astrocytes and neurons

**Fig. 7:**
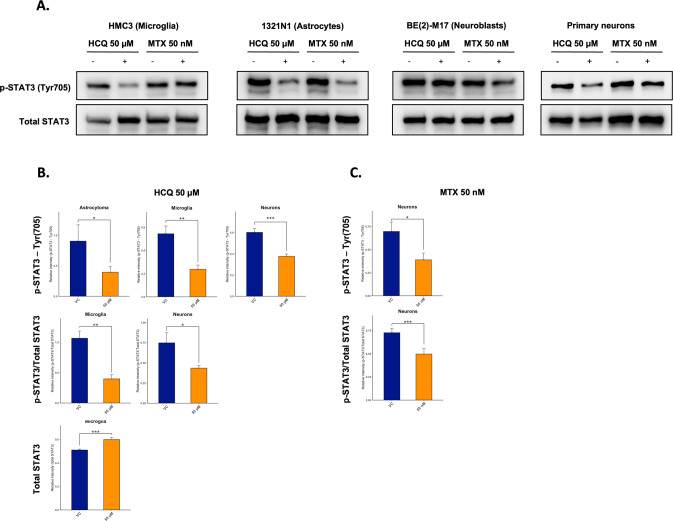
Hydroxychloroquine inactivates STAT3 in microglia, astrocytes and neurons. Human microglia cells, human astrocytoma cells, neuroblastoma cells, and mouse primary cortical neurons were either left untreated (VC) or treated with HCQ (50 µM, 48 h) or MTX (50 nM, 48 h), and the levels of p-STAT3 (Tyr705; primary epitope) and total STAT3 were assessed by western blot analysis, quantified by densitometry and normalized to levels of the loading control protein, ACTB. **A** Representative western blot images comparing treatment of HCQ or MTX (+) to VC (−). **B** After HCQ treatment, levels of p-STAT3 (Tyr705) were significantly reduced compared to the VC in astrocytes, microglia, and mouse primary cortical neurons; the ratio of p-STAT3/total STAT3 was significantly reduced in in microglia and mouse primary cortical neurons; HCQ did not have an impact on total STAT3 levels other than a significant increase in levels in microglia. **C** After MTX treatment, levels of p-STAT3 (Tyr705) and the ratio of p-STAT3/total STAT3 were significantly reduced compared to the VC. Error bars in all bar graphs indicate group mean + standard deviation (SD). Group differences comparing HCQ or MTX -treated cells to untreated control cells were evaluated using the one-way ANOVA test. Asterisks indicate significant differences between groups: *p < 0.05; **p < 0.01; ***p < 0.001. p-STAT3 phosphorylated STAT3, HCQ hydroxychloroquine, MTX methotrexate, Tyr705 tyrosine 705, ACTB β-Actin, VC vehicular control.

We tested whether HCQ treatment (48 h; 50 µM) compared to the vehicular control (VC) alters levels of total STAT3, phosphorylated STAT3 (p-STAT3; Tyr705, the primary phosphorylated epitope) and the ratio of p-STAT3/ total STAT3 in microglia, astrocytes, neuroblasts and neurons. HCQ significantly reduced p-STAT3 levels in astrocytes, microglia, and mouse primary cortical neurons; HCQ did not have an impact on total STAT3 levels other than a significant increase in levels in microglia; HCQ significantly reduced the ratio of p-STAT3/total STAT3 in microglia and mouse primary cortical neurons. Results were robust to distributional assumptions. Representative western blot images and bar plots visualizing significant results are included in Fig. 7A, B. Full western blot results are included in Supplementary Fig. 4 and a summary of all statistical results are included in Supplementary Table 6.

We additionally tested the effect of MTX treatment (48 h.; 50 nM). MTX significantly reduced p-STAT3 levels compared to the VC only in mouse primary cortical neurons; MTX did not have a significant effect on total STAT3 levels; MTX significantly reduced the ratio of p-STAT3/total STAT3 in mouse primary cortical neurons (Fig. 7C; Supplementary Fig. 4; Supplementary Table 6).

### HCQ-induced rescue of impaired hippocampal synaptic plasticity is associated with inactivation of STAT3

To test whether the HCQ-induced rescue of impaired hippocampal synaptic plasticity (described previously) may be associated with inactivation of STAT3, we tested levels of total STAT3 and phosphorylated STAT3 (p-STAT3; Tyr705 the primary phosphorylated STAT3 epitope) after treatment with HCQ (25 and 50 µM) in WT and APP/PS1 mouse hippocampal slices. Total STAT3 and p-STAT3 levels were significantly higher in untreated APP/PS1 hippocampi relative to WT; there was no change in the p-STAT3/total STAT3 ratio. After treatment of APP/PS1 hippocampi with 25 µM HCQ, we did not observe an inactivation of STAT3. Total STAT3 and p-STAT3 levels of treated and untreated APP/PS1 hippocampi were significantly higher compared to WT and there was no significant difference in total STAT3 and p-STAT3 levels comparing treated and untreated APP/PS1 hippocampi. There was also no change in p-STAT3/total STAT3 ratio after treatment of APP/PS1 hippocampi with 25 µM HCQ.

**Fig. 8:**
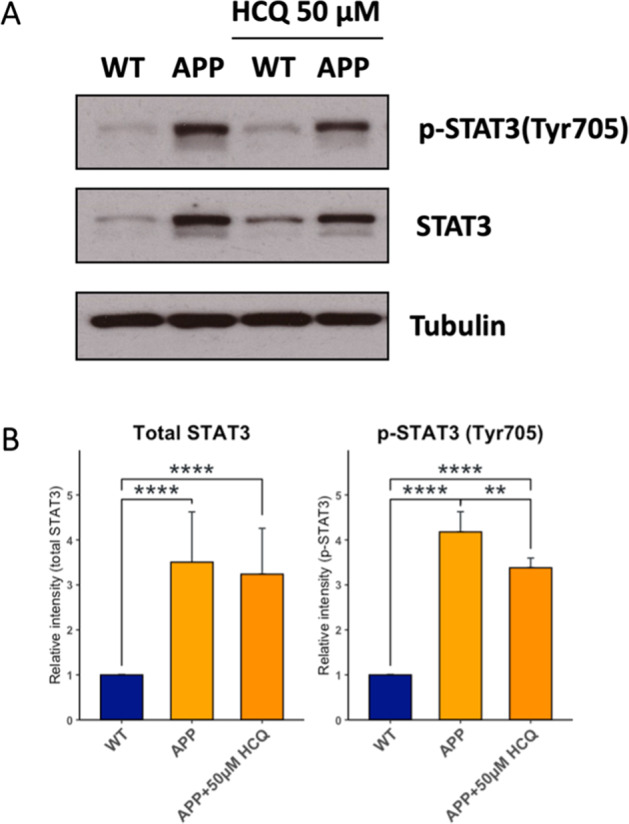
Hydroxychloroquine rescue of impaired late long-term potentiation is associated with inactivation of STAT3. WT and APP/PS1 mouse hippocampal slices were either left untreated or treated with HCQ (50 µM, 48 h), and the levels of p-STAT3 (Tyr705; primary epitope) and total STAT3 were assessed by western blot analysis, quantified by densitometry and normalized to levels of the loading control protein, tubulin and reported in comparison with WT. **A** Representative western blot images comparing total STAT3 and p-STAT3 levels across WT, APP/PS1, WT + 50 µM HCQ and APP/PS1 + 50 µM HCQ (*n* = 3 mouse hippocampi/group). **B** Levels of both total STAT3 and p-STAT3 were significantly higher in untreated and treated (50 µM HCQ) APP/PS1 hippocampi compared to WT. Levels of p-STAT3 were significantly lower in APP/PS1 + 50 µM HCQ-treated hippocampi compared to untreated APP/PS1 mice, while no differences in total STAT3 levels were observed. Error bars in all bar graphs indicate group mean + standard deviation (SD). Group differences were evaluated using the one-way ANOVA test. Asterisks indicates significant differences between groups: *p < 0.05, **p < 0.01, ***p < 0.001 and ****p < 0.0001). p-STAT3 phosphorylated STAT3, HCQ hydroxychloroquine, Tyr705 tyrosine 705, APP/PS1 double transgenic mice expressing AD pathology (human amyloid precursor protein mutant human presenilin 1), WT wild type mice.

After treatment of APP/PS1 hippocampi with 50 µM HCQ, we observed inactivation of STAT3. While total STAT3 and p-STAT3 levels were still significantly higher compared to WT, p-STAT3 levels were significantly lower in treated APP/PS1 hippocampi compared to untreated while total STAT3 levels remained unchanged (Fig. 8). There was no change in the p-STAT3/total STAT3 ratio. Complete results of the western blot analyses including all pair-wise comparisons are included in Supplementary Table 7.

## Discussion

Extending our recent work examining candidate AD treatments targeting the JAK/STAT cytokine signaling pathway [3, 9], we now demonstrate that HCQ lowers the incidence of ADRD compared to MTX in older individuals, rescues impaired hippocampal synaptic plasticity in APP/PS1 mice and corrects multiple molecular abnormalities underlying AD. Together, these findings suggest that HCQ may be a promising disease-modifying AD treatment in at-risk individuals.

We first tested whether exposure to HCQ lowers ADRD risk in humans in a large real-world clinical dataset. We implemented a rigorous study design that addresses several common pitfalls in pharmacoepidemiologic studies of ADRD including the lack of an active comparator group for the same indication, misclassification of ADRD onset, as well as lack of outcome specificity [3]. Across all prespecified analyses, we found that exposure to HCQ in older individuals prior to ADRD diagnosis was associated with 8–16% lowering of incident ADRD relative to the active comparator, MTX. These results were consistent when restricting the outcome to only AD as well as when using an alternate active comparator drug (i.e., leflunomide).

We next proceeded to test the effects of HCQ across molecular abnormalities underlying AD including impaired hippocampal synaptic plasticity which is believed to mediate cognitive impairment in AD. We show that HCQ restores late LTP [40, 41] in the hippocampus of APP/PS1 mice prior to significant accumulation of amyloid plaques and neurodegeneration. This restoration is dose-dependent, including a partial rescue at 25 µM HCQ and a complete rescue of impaired hippocampal synaptic plasticity at 50 µM HCQ. The comparator drug, MTX, did not show a similar rescue of late LTP. These findings are the first to suggest that HCQ may ameliorate dysfunction in the neural basis of learning and memory processes [42, 43] that may underlie neurocognitive impairment in AD [44–46].

We conducted additional exploratory analyses to test the effects of HCQ in phenotypic assays reflecting molecular features of AD pathogenesis to further assess its potential as a candidate AD treatment. Our results suggest that HCQ impacts key cellular functions relevant to AD pathophysiology [47, 48]. These include countering neuroinflammation by lowering release of pro-inflammatory cytokines in both BV-2 and adult 5xFAD microglial cells (Fig. 5A), enhancing the microglial clearance of extracellular Aβ_1-42_ through phagocytosis into acidic cellular compartments in both BV-2 and iPSC derived adult human AD microglial cells (Figs. 5B, 6) and reducing tau phosphorylation in neuroblastoma cells overexpressing human mutant tau (Fig. 5C).

Our findings suggest that HCQ lowers risk of incident ADRD compared to MTX and rescues abnormalities associated with AD including impaired hippocampal synaptic plasticity as well as the three principal pathogenic mechanisms in AD: neuroinflammation, Aβ clearance and tau phosphorylation. One potential mechanism explaining these findings may be through the inactivation of the cytokine transducer protein, STAT3. Recent findings suggest that HCQ inactivates STAT3 [11] while prior work in transgenic AD mouse models has implicated enhanced STAT3 signaling in Aβ-induced neuronal death, reactive astrogliosis, impaired microglial clearance of Aβ as well as in cognitive impairment [49, 50], suggesting that inhibition of STAT3 signaling may target multiple molecular abnormalities and hence present a novel therapeutic approach in AD. To assess whether STAT3 inactivation may be associated with disease-modifying effects of HCQ, we first showed that HCQ inactivates STAT3 in astrocytes, microglia, and mouse primary cortical neurons. We additionally showed that treatment of APP/PS1 hippocampi with HCQ significantly reduces p-STAT3 levels suggesting that the rescue of impaired hippocampal synaptic plasticity by may be associated with the inactivation of STAT3. These mechanistic studies, while preliminary suggest that HCQ effects on ADRD may be mediated through STAT3 inactivation.

A previous small (HCQ group: *N* = 77, placebo group: *N* = 78) clinical trial of HCQ in patients with established AD (i.e., symptomatic individuals) [51] in 2001 did not show a significant effect in slowing cognitive decline. Several methodologic issues in this previous clinical trial merit consideration. First, this trial was likely under-powered to show differences on cognitive endpoints between drug and placebo. In comparison, recent phase 2/3 clinical trials of amyloid-lowering drugs have recruited more than 1000 patients randomized to drug or placebo groups [52]. Additionally, prior bioavailability studies have shown that steady state levels of HCQ are only achieved after approximately six months of dosing [53, 54]. In a recent phase-2 clinical trial testing HCQ in patients with primary progressive multiple sclerosis, a run-in period of six months from the initiating treatment was incorporated and primary outcomes measured between 6 and 18 months [55]. The absence of a similar run-in period to achieve steady state HCQ levels in the prior 2001 clinical trial in AD may have further reduced the likelihood of detecting changes in clinical outcomes. Finally, the previous clinical trial of HCQ was performed well before the recognition of preclinical AD as a diagnostic entity and before the advent of biomarkers to accurately diagnose AD for patient recruitment into clinical trials. Interestingly, a recent case report showed that HCQ treatment in a patient diagnosed with sarcoidosis and mild cognitive impairment due to AD was associated with significant improvement in cognitive performance and accompanying correction of abnormal CSF Aβ_1-42_ levels [56].

Previous epidemiologic studies have examined the impact of HCQ on AD risk. Two such studies utilizing data from primary care patients in the UK and Taiwan showed no reduction in AD risk with HCQ treatment [57, 58]. Notably, our study differs substantially from these prior investigations as we explicitly compared the risk of ADRD in RA patients treated with HCQ or an equivalent alternative treatment (MTX) to minimize confounding by indication after accounting for a large number of potential confounding factors. Further, our study used a large cohort of HCQ treated patients and may therefore have had greater statistical power to detect smaller effect sizes.

Several features make HCQ an attractive candidate for repurposing in AD including its permeability across the blood brain barrier and effective partitioning into the brain. Doses of HCQ of 6.0–6.5 mg/kg/day typically used in RA patients, yield serum concentrations of 1.4 to 1.5 micromolar [59]. Brain concentrations are several fold higher than plasma and accumulation is likely even higher in acidic compartments including lysosomes [60, 61]. The doses tested in our in vitro experiments (i.e., 25 µM and 50 µM) may be consistent with brain concentrations achievable with conventional dosing of HCQ in RA patients. Furthermore, HCQ has a well-established safety profile with serious side effects being relatively rare [62], although additional screening for cardiac arrhythmias in some patients may be necessary [63, 64].

This study has limitations. First our phenotypic studies used a variety of cells lines to assess the effects of HCQ on several distinct AD-related phenotypes. These cell culture based phenotypic assays only reflect discrete aspects of AD pathogenesis are not capable of recapitulating complex gene-environment interactions that underlie the disease in older individuals. Second, we do not include cognitive data in our transgenic AD mouse models. While our pharmacoepidemiologic evidence suggests clinical benefits in humans, additional behavioral data from animal models testing the effects of HCQ would provide important experimental evidence for any potential benefit. This would be an important next step in follow-up studies. Third, our experiments to test whether STAT3 inactivation may be associated with disease-modifying effects HCQ are preliminary; the associations reported only suggest that HCQ may impact AD pathogenesis through this mechanism and merit confirmation in future studies.

In summary, we have established that the commonly used RA drug, HCQ lowers AD risk in older individuals, and targets multiple pathogenic mechanisms in AD including synaptic dysfunction, neuroinflammation, Aβ clearance, and tau phosphorylation. Our results provide compelling evidence that this safe and inexpensive drug may be a promising disease-modifying treatment for AD. Confirmation of our findings in adequately powered clinical trials in at-risk individuals during preclinical stages of disease progression should be initiated in a timely manner.

## Supplementary information

Supplementary Text

Supplementary Figures and Tables

## Data Availability

Aetion Evidence Platform v4.32 is designed with a point and click user interface that eliminates the need for line programming. All ICD, NDC, and procedure codes used to define our variables are available in the protocol registered at clinicaltrials.gov (https://clinicaltrials.gov/ct2/show/NCT04691505).
