# Supplementary material for: Hydroxychloroquine lowers Alzheimer’s disease and related dementias risk and rescues molecular phenotypes related to Alzheimer’s disease
Source: Mol Psychiatry. 2022 Dec 28;28(3):1312–26. doi: 10.1038/s41380-022-01912-0 (PMC10005941; doi:10.1038/s41380-022-01912-0)
Supplement: Supplementary file 1 — Supplementary Text [file 41380_2022_1912_MOESM1_ESM.docx]

**Supplementary Text**

**METHODS**

**HCQ ameliorates AD-related phenotypes in cell culture**

*Cultivation and isolation of MACS adult 5xFAD microglia (for Aβ_1-42_ Clearance and Lipopolysaccharide (LPS)-induced neuroinflammation assays)*

Adult microglia were isolated from 9 months old 5xFAD mice via magnetic cell sorting (MACS). Therefore mice were terminally anesthetized by i.p. injection of Pentobarbital (600 mg/kg, dosing 10 μl/g body weight) and brains were transcardially perfused with DPBS. Brains were removed, the brainstem discarded and the remaining brain minced for cell dissociation. Cell dissociation was performed using Miltenyi Adult Brain Dissociation Kit (Miltenyi, 130-107-677). MACS cell separation was performed using CD11b (Microglia) MicroBeads, mouse (miltenyi, 130-093-634) and MS columns on OctoMACS cell separator (Miltenyi). Isolated microglia were seeded onto 0.01% PLL coated plates at a density of 10.000 cells per well in 384 well plate in DMEM containing 10% FBS,1% penicillin/streptomycin, and 2 mM L-glutamine.

*Cultivation of AD iPSC derived microglia cells (for Aβ_1-42_ Clearance)*

Microglia derived from human induced pluripotent stem cells were purchased as kit from FUJIFILM Cellular Dynamics Inc (CatNo 1212, Female, APOE 4/4, Alzheimer's Disease) and maintained according to the provided manual. Cells were seeded at a density of 10.000 cells per well in 384 well plate in the kit provided media.

*Lipopolysaccharide (LPS)-induced neuroinflammation – BV-2 microglia*

The murine microglial cell line BV-2 was cultivated in DMEM medium supplemented with 10% FCS, 1% penicillin/streptomycin and 2 mM L-glutamine (culture medium). For LPS stimulation assay, 5000 BV-2 cells per well (uncoated 96 well plates) were plated out and the medium was changed to treatment medium (DMEM, 5% FCS, 2 mM L-glutamine). After changing cells to treatment medium, HCQ (25, 2.5 and 0.25 µM) was applied 1 hour before LPS stimulation (Sigma-Aldrich; L6529; 1 mg/ml stock in ddH_2_O, final concentration in well: 100 ng/mL (dilutions in medium)). Cells treated with vehicle, cells treated with LPS alone, as well as cells treated with LPS plus reference item (dexamethasone 10 µM, Sigma D4902) served as controls. After 24 h of stimulation, cell supernatants were collected for cytokine measurements (V-PLEX Proinflammatory Panel 1 Mouse Kit, K15048D, Mesoscale) and MTT assay.

*LPS induced neuroinflammation – MACS isolated adult 5xFAD microglia*

On DIV7 HCQ at 0,.25 µM, 2.5 µM and 25µM was applied 1 hour before LPS stimulation (Sigma-Aldrich; L6529; 1 mg/ml stock in ddH2O, final concentration in well: 50 ng/mL (dilutions in medium)). Cells treated with vehicle, cells treated with LPS alone, as well as cells treated with LPS plus reference item (dexamethasone 10 µM, Sigma D4902) served as controls. After 24 h of stimulation, cell supernatants were collected for the cytokine measurement (V-PLEX Proinflammatory Panel 1 Mouse Kit, K15048D, Mesoscale).

*Aβ*_1-42_ *Clearance – BV-2 microglia*

For Abeta (Aβ_1-42_) clearance assay, 20,000 BV-2 cells per well (uncoated 96 well plates) were plated out. After changing cells to treatment medium, HCQ at 25, 2.5 and 0.25 µM was applied 1 hour before Aβ_1-42_ stimulation (Bachem 4061966; final concentration in well: 200 ng/mL (dilutions in medium)). Cells treated with vehicle (H_2_O) and cells treated with Aβ_1-42_ alone served as controls. After 3 h of Aβ_1-42_ stimulation, cell supernatants were collected for the Aβ_1-42_ measurement and cells were carefully washed twice with PBS and thereafter lysed in 35 µL cell lysis buffer (50 mM Tris-HCl, pH 7.4, 150 mM NaCl, 5 mM EDTA, 1% SDS) supplemented with protease inhibitors. Supernatants and cell lysates were analyzed for human Aβ_1-42_ with MSD® V-PLEX Human Aβ42 Peptide (6E10) Kit (K151LBE, Mesoscale Discovery). The assay was carried out according to the manufacturer’s manual and plates were read on the MESO QuickPlex SQ 120.

*Aβ1-42 Clearance - MACS isolated adult 5xFAD microglia and AD iPSC derived microglia cells*

On DIV7 HCQ at 25 µM, 2.5 µM and 0.25 µM was applied 1 hour before Aβ1-42 stimulation (Bachem 4061966; final concentration in well: 4 µM (dilutions in medium) labelled with pHrodo Red (Thermo Fisher, P36011). Cells treated with vehicle (H2O) and cells treated with Aβ1-42 alone served as controls.

After 4 h and 24 h of Aβ1-42 stimulation, cell supernatants were collected for the Aβ1-42 measurement. Supernatants were analyzed for human Aβ1-42 with MSD® V-PLEX Human Aβ42 Peptide (6E10) Kit (K151LBE, Mesoscale Discovery). The immune assay was carried out according to the manufacturer’s manual and plates were read on the MESO QuickPlex SQ 120.

At the same time points (4h and 24h after Aβ1-42 addition) images were acquired using IncuCyte® (Sartorius) in brightfield and RFP channel to monitor intracellular pHrodo red signal increase. Data were evaluated as integrated RFP intensity per µm^2^.

*Aβ*_1-42_ *Clearance-Protonex assay*

For Abeta (Aβ_1-42_) clearance Protonex assay, 5000 BV-2 cells per well (uncoated 96 well plates) were plated out. After changing cells to treatment medium, HCQ was applied 1 hour before stimulation with Protonex™ Green 500, SE (21216, AAT Bioquest)-labelled Aβ_1-42_ (Bachem 4061966; final concentration in well: 200 ng/mL (dilutions in medium)). Cells treated with vehicle and cells treated with Aβ_1-42_ alone served as controls. After 3 h of Aβ_1-42_ stimulation, cells were imaged on Cytation 5 multimode reader (Biotek) and green fluorescence was measured.

*Aβ*_1-42_ *toxicity*

Primary hippocampal neurons were prepared from E18.5 timed pregnant C57BL/6JRccHsd mice as previously described. Cells were seeded in poly-D-lysine pre-coated 96-well plates at a density of 4x10^4^ cells/well and cultivated until DIV10 (Neurobasal, 2% B-27, 0.5 mM glutamine, 25 μM glutamate, 1% Penicillin-Streptomycin). On DIV10 pre-aggregated Aβ_1-42_ (Bachem 4061966, final concentration 10 µM, 48h at 4°C) was added to the cells in the presence or absence of Hydroxychloroquine sulfate (TargetMol, T0951) at 25, 2.5 or 0.25 µM concentrations. On DIV16 cells were subject to MTT assay to determine cell viability.

*Aβ secretion*

Human APP overexpressing H4-hAPP cells were cultivated in Opti-MEM supplemented with 10% FCS, 1% penicillin/streptomycin 200 µg/mL Hygromycin B and 2.5 µg/mL Blasticidin S.

H4-hAPP cells were seeded into 96 well plates (2 x 10^4^ cells per well). The following day, cells in 96 well plates were treated HCQ (25, 2.5 and 0.25 µM) or the reference item (DAPT 400 nM), a γ-secretase inhibitor vehicle. 24 h later, supernatants were collected for further Aβ (Aβ_1-42_, Aβ_1-40_, Aβ_1-38_, Aβ_42:40_) measurements by MSD® (V-PLEX Aβ Peptide Panel 1 (6E10) Kit, K15200E, Mesoscale Discovery).

*Tau phosphorylation*

SH-SY5Y-hTau441(V337M/R406W) cells were maintained in culture medium (DMEM medium, 10% FCS, 1% NEAA, 1% L-Glutamine, 100 µg/mL Gentamycin, 300 µg/mL Geneticin G-418) and differentiated with 10 µM retinoic acid (RA) for 5 days, changing the medium every 2 to 3 days. Prior to the treatment, cells were seeded onto 24-well plates at a cell density of 2 x 10^5^ cells per well (DIV1). HCQ (25, 2.5 and 0.25 µM) was applied on DIV2. After 24 h of incubation (DIV3), cells on 24-well plates were harvested in 60 µL RIPA-Buffer [50 mM Tris pH 7.4, 1% Nonidet P40, 0.25% Na-deoxy-cholate, 150 mM NaCl, 1 mM EDTA supplemented with freshly added 1 µM NaF, 0.2 mM Na-ortho-vanadate, 80 µM Glycerophosphate, protease (Calbiochem) and phosphatase (Sigma) inhibitor cocktail]. Protein concentration was determined by BCA assay (Pierce, Thermo Fisher) and samples were adjusted to a uniform total protein concentration. Total Tau and phosphorylated Tau were determined by immunosorbent assay from Mesoscale Discovery (Phospho(Thr231)/Total Tau Kit K15121D, Mesoscale Discovery).

*Cell death due to trophic factor withdrawal*

Primary cortical neurons from E18 C57Bl/6 mice were prepared as previously described. On the day of preparation (DIV1), cortical neurons were seeded on poly-D-lysine pre-coated 96-well plates at a density of 3 x 10^4^ cells per well. Every 4-6 days, a half medium exchange using full medium (Neurobasal, 2% B-27, 0.5 mM glutamine, 1% Penicillin-Streptomycin) was carried out. On DIV8, a full medium exchange to B-27 free medium (Neurobasal, 0.5 mM glutamine, 1% Penicillin-Streptomycin) was performed and HCQ (25 µM, 2.5 µM and 0.25 µM) was applied thereafter. The experiment was carried out with n=6 technical replicates per condition, vehicle treated cells served as control. After 28 h on B-27 free medium, cells were subject to YO-PRO-PI and MTT as well as LDH assay.

YO-PRO™-1 (Invitrogen; Y3603) assay was carried out to detect apoptotic cells in combination with Propidium iodide (PI; P4864 Sigma Aldrich) staining for necrotic cells.

Part of the supernatant of the cultivated cells was aspirated, so that 90 μL remained per well. 50 μM YO-PRO 1 solution was prepared out of the 1 mM YO-PRO 1 stock solution in DMSO. The stock solution was diluted in a ratio of 1:20 in PBS and Propidium iodide (PI) was added to the same stock to a final concentration of 1 µg/mL. 10 μL of this 50 μM YO-PRO 1/1µg/mL PI solution in PBS was added to the remaining 90 μL to result in a final concentration of 5 μM YO-PRO-1 in the well. Incubation for 15 min in the incubator at 37°C was performed (light protected). Supernatant was aspirated completely and discarded. 140 μL PBS was added to well. The plate was measured using a multimode-reader (Cytation 5, BioTek) at 485_EX_/535_EM_.

MTT solution was added to each well in a final concentration of 0.5 mg/mL. After 2 h, the MTT containing medium was aspirated. Cells were lysed in 3% SDS and the formazan crystals were dissolved in isopropanol/HCl. Optical density was measured with a Cytation 5 (Biotek) multimode reader at wavelength 570 nm. Values were calculated as percent of control (vehicle control or lesion control).

The Lactate dehydrogenase (LDH) toxicity assay was carried out on the supernatants collected after treatment using the Cytotoxicity Detection Kit (Roche Diagnostics, Cat. No: 11 644 793 001). 70 μL of cell culture supernatant was transferred to clear 96-well plates. 70 μL freshly prepared reaction mixture was added to each well and the mixture was incubated for 1 h at room temperature protected from light. Absorbance was measured at 492 nm and 620 nm as reference wavelength with a Cytation 5 (Biotek) multimode reader. Values of culture medium were subtracted as background control. Values were calculated as percent of control (VC or lesion control).

*Neurite Outgrowth and neurogenesis*

Primary hippocampal neurons were prepared from E18.5 timed pregnant C57BL/6JRccHsd mice as previously described. Cells were seeded in poly-D-lysine pre-coated 96-well plates at a density of 2.6x104 cells/well in medium (Neurobasal, 2% B-27, 0.5 mM glutamine, 25 μM glutamate, 1% Penicillin-Streptomycin). Directly on DIV1, HCQ (TargetMol, T0951) at 25 µM, 2.5 µM or 0.25 µM concentrations or VC was applied. On DIV2, 10 µM Bromodeoxyuridine (BrdU; B5002 Sigma Aldrich) was added and cells were fixed after additional 24h. Cells were permeabilized with 0.1% Triton-X and incubated with primary Beta Tubulin Isotype III (T8660, Sigma Aldrich) and BrdU antibodies (MAS250c, AHrlan-Sera Lab) overnight at 4°C. Afterwards cells were washed two times with PBS and incubated with fluorescently labeled secondary antibodies and DAPI for 1.5 hour at RT in the dark. Cells were rinsed three times with PBS and imaged with the Cytation 5 Multimode reader (BioTek) at 10x magnification (6 images per well). BrdU positive cells were counted as a marker of neurogenesis and Beta Tubulin Isotype III signal was used for macro-based quantification of neurite outgrowth.

**RESULTS**

**HCQ rescues impaired hippocampal synaptic plasticity in APP/PS1 mice**

In order to test the effect of MTX (50 nm) in WT mice, late-LTP was induced by STET in synaptic input S1, in presence of MTX which resulted in a potentiation that remained stable for 180 min (filled blue circles; n=7, Supplementary Figure 3A). We observed statistically significant differences in field excitatory postsynaptic potentials (fEPSP) from 1 min until 180 min when compared with its own baseline and with control input S2 (*p*<0.05 using Wilcoxon test and Mann-Whitney U-test). A comparison of late-LTP - WT S1 (Figure 4B) with WT + MTX S1 (Supplementary Figure 3A) did not show any significant differences at any compared timepoints (1 min, p=0.21, 60 min, p=0.38,120 min p=0.90 and 180 min p= 0.99).

We then tested whether MTX (50 nM) had any effect on late-LTP in hippocampal slices from APP/PS1 mice. As shown in Supplementary Figure 3B, bath-application of MTX, 30 min before and 30 min after the STET induced only a short lasting LTP in the APP/PS1 hippocampus (filled blue circles; n=7). Significant differences were observed in fEPSP only until 120 min, when compared to its own baseline and until 105 min when compared to S2 (120 min Wilcox, p=0.04, 115 min U-test, p=0.02 respectively). A comparison of late-LTP – APP/PS1 S1 (Figure 4C) with APP/PS1 + MTX S1 (Supplementary Figure 4B) did not show any significant differences at any compared timepoints (1 min, p=>0.99, 60 min, p=0.73, 120 min p=0.73 and 180 min p=0.45).
